# Transnasal Endoscopic Treatment of Tension Pneumocephalus Caused by Posttraumatic or Iatrogenic Ethmoidal Damage

**DOI:** 10.1155/2023/2679788

**Published:** 2023-08-22

**Authors:** Goran Latif Omer, Riccardo Maurizi, Beatrice Francavilla, Kareem Rekawt Hama Rashid, Gianluca Velletrani, Hasan Mustafa Salah, Giulia Marzocchella, Mohammed Ibrahim Mohialdeen Gubari, Stefano Di Girolamo

**Affiliations:** ^1^Department of Surgery, University of Sulaimani, College of Medicine, Sulaymaniyah, Iraq; ^2^Department of Otorhinolaryngology, University of Rome Tor Vergata, Rome 00133, Italy; ^3^Department of Otorhinolaryngology, Royal Hospital, Sulaymaniyah, Iraq

## Abstract

**Background:**

Tension pneumocephalus is a neurosurgical emergency caused by progressive accumulation of air in the intracranial spaces mediated by a valve mechanism. Tension pneumocephalus usually presents with headaches, reduced consciousness, and even death. One of the most common causes is an ethmoidal defect resulted by nasal surgery or facial traumas.

**Methods:**

A literature review about tension pneumocephalus resulting from ethmoidal damages was performed. Surgery strategies included decompression by frontal burr holes and multilayer repair of the ethmoidal defect. In this paper, an endoscopic technique that exploits the ethmoidal defect to decompress the intracranial spaces and to resolve tension pneumocephalus with fewer complications and shorter hospitalization in comparison to frontal craniotomy is proposed.

**Conclusion:**

The proposed endonasal endoscopic technique could be effectively used as a first-line treatment for symptomatic tension pneumocephalus caused by posttraumatic or iatrogenic ethmoidal defect.

## 1. Introduction

Pneumocephalus is defined as the presence of cranial cavity air, often associated with neurosurgery, endoscopic sinus surgery, and craniofacial trauma. If the air within the intracranial cavity becomes trapped by a “ball-valve” or “inverted pop bottle” mechanism, increased intracranial pressure may cause tension pneumocephalus (TP), with consequent mass effect and neurologic deterioration [1, 2]. Simple pneumocephalus is mostly asymptomatic, and it may resolve spontaneously. However, TP is a surgical emergency, and active management is essential.

Due to the multifactorial etiology and diversified therapeutic approaches, there is no consensus regarding treatment of TP [3]. While open repair of the defect is the historical standard, recent reports described efficacious transnasal endoscopic repair of the skull base defect and the cerebrospinal fluid (CSF) leak, which is best approached using multilayered techniques [3–6]. Once the identification of the skull base defect has been achieved, the need for invasive surgical decompression must be determined [3]. For cases of simple pneumocephalus, decompression is rarely performed, and the intracranial air is gradually reabsorbed after skull base defect closure. TP more often requires neurosurgical decompression, particularly when its evolution is rapid and life-threatening [2].

In this paper, a novel endoscopic transnasal approach for the endonasal treatment of tension pneumocephalus with CSF leak is proposed. The described technique combines endoscopic multilayer repair and decompression by transnasal normal saline irrigation through the ethmoidal breach. No written consent has been obtained from the patient as there are no patient identifiable data included in this case report.

## 2. Case Report

A 55-year-old man with a history of noneosinophilic nasal polyposis underwent functional endoscopic sinus surgery (FESS) in another hospital. Nasal packing was removed in the 3th postoperative day, and after that, the patient developed clear right rhinorrhea and mild headache. 12 hours later, the patient was transferred to the otorhinolaryngology department of our hospital presenting with severe headache, bradycardia, nausea, vomiting, and altered state of consciousness (Glasgow Coma Scale 8).

According to the neurosurgeon, an urgent CT scan was performed, showing sever extensive pneumocephalus with the right-side skull base defect at the right-side lateral lamella of the cribriform plate.

## 3. Surgical Technique

**Figure 1:**
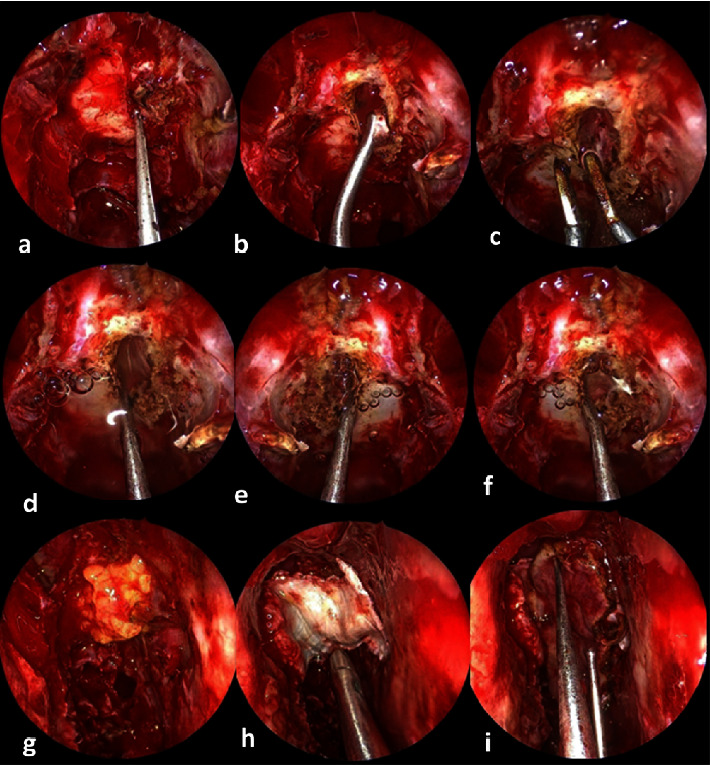
(a) Anterior skull base defect exposure; (b) enlargement of the breach to remove valve action and sharp edges; (c) cauterizing small bleeding vessels surrounding the bone rupture; (d–f) instillation of warmed normal saline. Note that the air bubble blowing out: (g) sealing the defect with a thigh fat plug, (h) fascia lata insertion, and (i) modified Hadad flap.

Our proposal of intervention provided, in a single surgical stage, both resolution of TP and closure of the bone defect that leads to cerebrospinal fluid leak. The patient lay in the reverse Trendelenburg position with the head elevated higher than the feet by 30°. The operative approach was by transnasal endoscopy using a 0° angle, 18 cm length, and 4 mm diameter rigid videoendoscope (Karl Storz GmbH, Tuttlingen, Germany) in order to detect the anterior skull base defect. Breach enlargement was performed to remove valve action and to sharp edges of the defect, improving the chances of repair. Endoscopic haemostatic treatment with bipolar electrocoagulation was used to cauterize small bleeding vessels surrounding the bone rupture. Dissection of the dura from skull base bone was performed so that a Gelfoam segment was inserted between the dura and the brain to create a tunnel allowing the spillage of trapped air. Warmed normal saline (W-NS) at body temperature (37°C) was infused through the gap with a 20cc syringe and then aspirated with a small olive tip sucker. With the aid of the Valsalva maneuver performed by the anesthesiologist, air bubbles began to come out as W-NS replaced trapped air. This procedure was repeated several times for one hour until most of the air was released. At the beginning of the procedure, no brain movement was noted due to intracranial compression of the air. However, at the end of the procedure, the brain found space for his pulsation, helping further air outflow. Finally, a multilayer technique was performed to secure the defect using thigh fat plug, Surgicel, and fascia lata, covered by the modified Hadad nasoseptal flap (Figure 1).

During the first postoperative day, the Glasgow Coma Scale score was 15. In the second postoperative day, a new CT-scan was executed, showing resolution of radiologic TP signs. The patient was released on the 4th postoperative day in good general condition.

## 4. Discussion

Pneumocephalus (PNC), also known as pneumatocele or intracranial aerocele, is the cranial vault presence of air [7], usually indicating a breach in the craniodural barrier [3]. Most common causes include head trauma, infections, neurosurgical procedures, and ENT operations such as paranasal sinus surgery, nasal septum resection, or nasal polypectomy [8]. Simple pneumocephalus usually gets absorbed spontaneously over several days, without any clinical manifestations [2, 8].

TP is a rare and life-threatening condition determined by continuous intracranial air accumulation that cannot escape or be resorbed quickly, with a concurrent increase of intracranial pressure and progressive brain compression [9]. Altered mental status and headache are the most common presenting symptoms. Other symptoms include visual disturbances, dizziness, confusion, vomiting, focal neurologic deficits, and progressive numbness [10]. An urgent cranial CT-scan is the key for diagnosis and tempestive treatment. The Mount Fuji sign is a typical radiological finding in which frontal lobes are compressed by intracranial air, creating a twin peak appearance on axial views of a CT scan [11].

TP is an emergency requiring immediate intervention to prevent serious neurological complications or even death. A therapeutic strategy should depend on the mass effect and clinical symptoms. In patients with mild symptoms, conservative treatment including bed rest with head elevation and normobaric hyperoxia with 100% inspired oxygen may be initially chosen under close observation [8].

In TP patients presenting with severe symptoms, treatment involves emergent decompression to release intracranial pressure and to repair the causative defect. Urgent surgical multilayer repair is usually performed to the closure of the craniodural breach [3, 6]. Neurosurgical decompression options include needle aspiration, controlled decompression via a closed water-seal drainage system, ventriculostomy, trephination with the creation of cranial burr holes, and decompressive craniectomy [8, 12]. Craniotomy is an emergent neurosurgical procedure that leads to elevate risks of complications including soft tissue infection, extradural abscesses, subdural empyema, bone flap infection, and postoperative intracranial infection [13]. Infection may manifest as several different ways, including formation of subdural empyema, intraaxial or extraaxial abscesses, and meningitis, with an overall postcraniotomy intracranial infection incidence of 6.8% [14].

In a recent literature review, Dalolio et al. described the endoscopic endonasal approach as an effective second-line treatment of symptomatic posttraumatic TP [15]. Li et al. [9] reported 26 cases of TP caused by endoscopic endonasal surgery collected in an English literature review: 11 cases were caused by an ethmoidal defect, and the therapeutic approach was based on a defect multilayer closure with or without craniotomy.

A review of the English literature was performed, researching cases of TP associated with an ethmoidal CSF leak(Table 1). 21 TP cases associated with iatrogenic or posttraumatic isolated ethmoidal defect have been collected. The mean age was 59. 2 years, and the male/female ratio was about 3 : 1. The most frequent causes were endoscopic surgery for nasal polyposis (4 cases), septoplasty (4 cases), and facial trauma with ethmoidal plate fracture (4 cases). One case of TP developed after balloon sinuplasty for chronic rhinosinusitis and one case after Graves's disease surgery. In 12 cases, craniotomy with frontal burr holes was performed: 10 of those cases were associated with multilayer defect repair and 1 case with lumbar drain. Seven cases were treated by a multilayer repair of the ethmoidal defect without craniotomy and 2 cases with a conservative approach.

This paper describes a novel and effective technique that proposes a transnasal approach not only for the multilayer repair of the craniodural ethmoidal breach but also for TP decompression. The novelty of this procedure is the normalisation of the intracranial pressure using the injection of W-NS through the ethmoidal defect, releasing air from the cranial cavity. W-NS fills all the intracranial spaces by replacing air bubbles, preventing brain collapse after air drainage. The endoscopic endonasal reconstruction of the ethmoidal defect may be performed using different materials, both autologous and nonautologous, individually or combined in a multilayer fashion. The multilayer repair with a nasoseptal flap is one of the most effective methods: it reinforces the skull base closure granting isolation of the surgical field [30].

The main advantage of endoscopic transnasal approach is the possibility of achieving a direct visualization of the ethmoidal breach, minimizing brain displacement and manipulation. Moreover, this procedure allows us to avoid the infectious and haemorrhagic complications of craniotomy. Patients treated with this strategy may benefit of reduced postoperative morbidity, early discharge, and faster return to work.

## 5. Conclusion

Characterized by the presence of increasing amounts of intracranial air and concurrent appearance of neurological symptoms, TP is a life-threatening condition that can be devastating if not recognized and treated promptly. In case of ethmoidal CSF leak, a transnasal endoscopic approach may be used for the simultaneous repair of the damage and decompression by the injection of W-NS through the ethmoidal defect. The proposed endonasal endoscopic technique could be effectively used as a first-line treatment for symptomatic TP caused by posttraumatic or iatrogenic ethmoidal defect.

## Figures and Tables

**Table 1:** Literature review research cases of TP associated with ethmoidal CSF leak.

Reference/year	Case #	Age (y)/sex	Primary diseases	Defect localization	Therapeutic approach to TP
Martínez-Capoccioni et al. 2014 [1]	1	49/M	—	Anterior ethmoid roof defect	Multilayer closure with a slice of cartilage and free middle turbinate mucosal graft, fibrin glue, and layers of Surgicel
	2	26/M	Nasal polyp	Ethmoid roof defect	Multilayer closure with a slice of septal cartilage, fibrin glue, and layers of Surgicel
	3	55/F	Nasal polyp	Cribriform plate defect	Cranial burr hole and multilayer closure with a slice of septal cartilage, fibrin glue, and Surgicel
	4	22/F	Nasal septum deviation	Skull base defect (back wall of the posterior ethmoid sinus)	Multilayer closure with free nasal septal perichondrium and cartilage grafts only, fibrin glue, and multiple layers of absorbable packing
Prüss et al. 2011 [16]	5	58/M	—	Cribriform plate defect	Release of intracranial tension, duraplastic procedure
Aksoy et al. 2013 [17]	6	53/F	Nasal polyp	Medial-medium part of the ethmoid roof defect	Frontal burr hole and multilayer closure with fascia lata and adipose tissue grafts
Clevens et al. 1994 [18]	7	62/M	Nasal polyp	Cribriform plate defect	Bifrontal craniotomy and lumbar drain
Whitmore et al. 2008 [19]	8	72/M	Chronic rhinosinusitis	Roof of the left ethmoid sinus	Multilayer closure with temporalis fascia graft, Avitene, Gelfoam, and a middle meatal spacer
Simmons and Luks 2013 [10]	9	89/M	—	Fovea ethmoidal bone defect bilateral	Cranial burr hole, multilayer closure
Wong et al. 2018 [20]	10	67/M	Nasal polyp	Defect of the lateral lamella and fovea ethmoidalis	Multilayer closure using synthetic inlay, an inferior turbinate-free mucosal graft onlay, and DuraSeal
Çelikoğlu et al. 2016 [21]	11	72/F	Nasal polyp	Anterior skull base defect	Bifrontal craniotomy and repair with galea and fibrin glue
Naraghi and Ghazizadeh 2012 [22]	12	52/M	Nasal septum deviation	Left cribriform plate defect	Cranial burr hole
Bly et al. 2014 [4]	13	89/M	Chronic rhinosinusitis	Ethmoid roof defect	Cranial burr hole, multilayer closure
McCormick et al. 2021 [23]	14	76/M	Chronic rhinosinusitis (balloon sinuplasty)	Left cribriform plate defect	Left-sided nasoseptal flap with fibrin sealant, Gelfoam, and finger cot
Çiçek and Tan 2021 [24]	15	—	Nasal septum deviation	Ethmoid roof defect	Conservative treatment
Dewaele et al. 2007 [25]	16	92/M	Trauma	Cribriform plate defect	Burr hole and multilayer closure with free autologous pericranium and fibrin glue
Zasler, 1999 [26]	17	26/M	Trauma	Right ethmoid fracture	Craniotomy and multilayer closure with fascia lata graft
Kim et al. 2022 [27]	18	81/M	Trauma	Left ethmoidal cribriform plate defect	Frontal burr hole and bone graft, a mucosal flap, and fibrin sealant
Chastanet et al. 2020 [28]	19	48/F	Nasal septum deviation	Cribriform plate	Conservative treatment
Lasoff et al. 2019 [29]	20	59/M	Graves' disease	Left cribriform plate defect	Craniotomy and multilayer closure
Dalolio et al. 2022 [15]	21	37/M	Trauma	Multiple skull base defect within the anterior ethmoid defect	Multilayer closure

## Data Availability

Data sharing is not applicable for this case report.
